# A case of adult‐onset Still's disease in a patient after a car accident

**DOI:** 10.1002/ccr3.7510

**Published:** 2023-08-21

**Authors:** Feride Yaman, Ali Kimiaei

**Affiliations:** ^1^ Department of Pulmonology Bahcesehir University School of Medicine İstanbul Turkey; ^2^ Bahcesehir University School of Medicine İstanbul Turkey

**Keywords:** adult‐onset Still's disease, fever of unknown origin, pleural effusion

## Abstract

**Key Clinical Message:**

Adult‐onset Still's disease is a rare inflammatory condition with diverse clinical features. Yamaguchi criteria aid diagnosis, and pleural effusion and elevated ferritin levels are important markers. Steroids are the first‐line treatment.

**Abstract:**

Adult‐onset Still's disease (AOSD) is a rare systemic inflammatory condition with an unknown etiology. It is characterized by, spiking fever, arthritis, evanescent rash, sore throat, serositis, hepatomegaly, splenomegaly, and lymphadenopathy. It is a diagnosis of exclusion and has infections, systemic autoimmune and inflammatory rheumatic diseases, malignancy, and adverse drug reactions as its differential diagnosis. Because of these characteristics, diagnosis is frequently delayed, posing a significant challenge for physicians. While several classification criteria can be used to diagnose Still's disease, they have limitations in terms of sensitivity and specificity. The Yamaguchi criteria are considered the most sensitive and commonly used, requiring the presence of at least five characteristics, with at least two being major diagnostic criteria. Steroid therapy is the first‐line treatment for AOSD patients. In this case report, we present a 56‐year‐old female patient who developed pleurisy a few months after a car accident, subsequently diagnosed with adult‐onset Still's disease.

## Graphical abstract

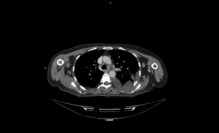

## 1 INTRODUCTION

Adult‐onset Still's disease (AOSD), a rare systemic inflammatory condition with an unknown etiology, is characterized by, spiking fever, arthritis, and evanescent rash. Sore throat, serositis, hepatomegaly, splenomegaly, and lymphadenopathy are additional often seen clinical characteristics.^1^ Laboratory findings in AOSD commonly show elevated ESR, leukocytosis (>10,000/mm^3^) with neutrophilic predominance, elevated AST and ALT, anemia, hyperferritinemia, and thrombocytosis.^2^ The incidence of this rare condition ranges from 0.16 to 0.4 per 100,000 people.^3^ Due to the diverse clinical presentations, the diagnosis of AOSD requires ruling out infectious, neoplastic, and autoimmune diseases among other differential diagnoses. Several diagnostic criteria have been proposed for AOSD, with the Yamaguchi criteria being the most sensitive, exhibiting a sensitivity of 93.5%.^4^ Major criteria include arthralgia lasting more than 2 weeks, fever higher than 39°C, typical rash, and white blood cell count greater than 10,000/mm^3^ with granulocyte predominance. Minor criteria include sore throat, lymphadenopathy and/or splenomegaly, abnormal liver function tests, and negative antinuclear antibody (ANA) and rheumatoid factor (RF) results. The diagnosis requires the fulfillment of at least five criteria, with at least two being major criteria.^5^

Corticosteroids and nonsteroidal anti‐inflammatory drugs (NSAIDs) are the first‐line treatments in patients with AOSD.^2^, ^6^ Due to its rarity, the majority of AOSD studies are based on single clinical reports or limited case series, which limits the generalizability of the findings. In this case report, we present a 56‐year‐old female patient who developed AOSD following a car accident.

## 2 CASE

**FIGURE 1:**
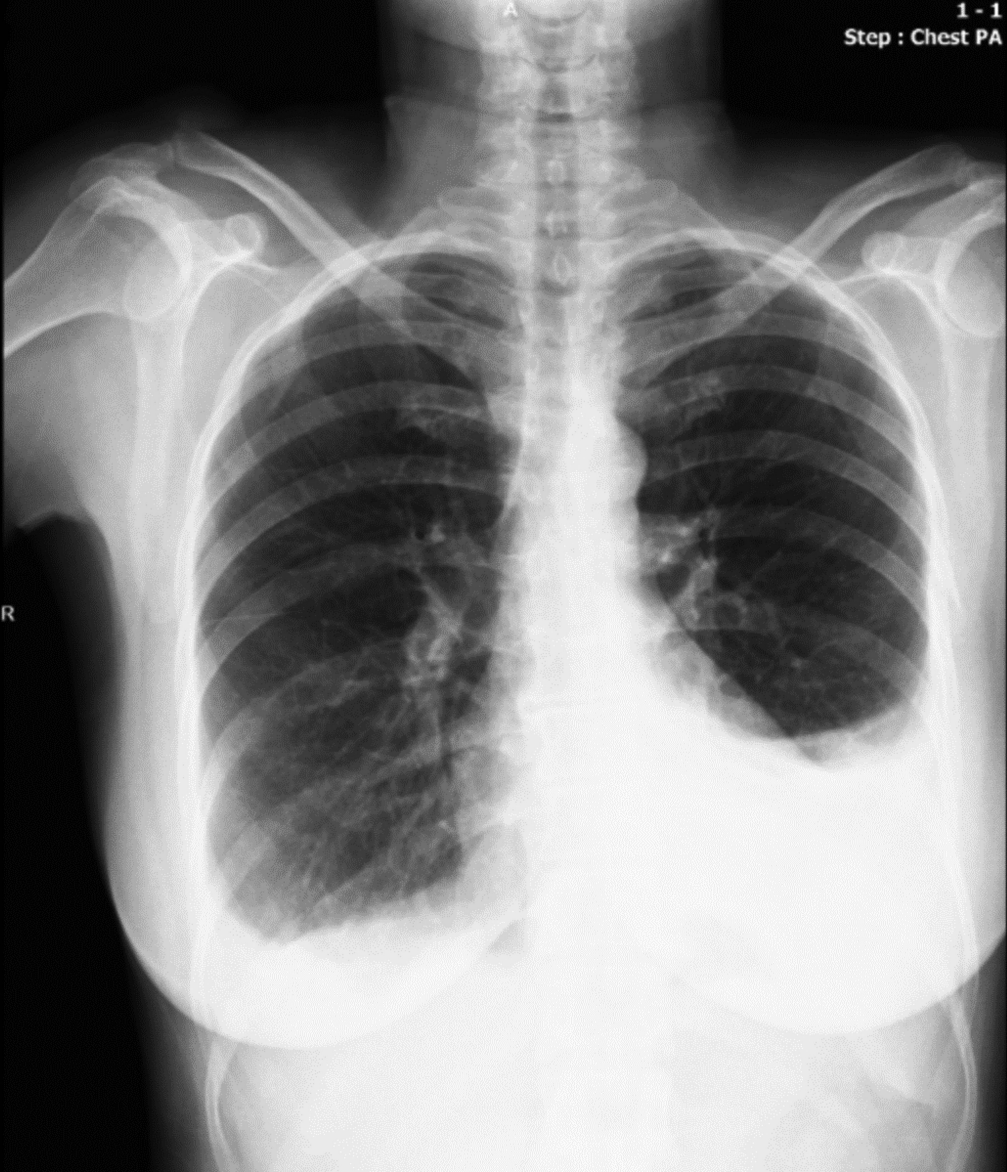
Bilateral pleural effusion, more prominent on the left, developed 1 month after the operation.

A 56‐year‐old healthy female with no significant past medical history was involved in a car accident in November 2021, resulting in surgical repair of a left diaphragmatic rupture. Approximately 1 month after the operation, she developed bilateral pleural effusion, with the left side being more prominent (Figure 1). The patient underwent thoracentesis and analysis of the fluid revealed exudative characteristics (protein: 3.9 g/dL, LDH: 205 μ/L, albumin: 1.7 g/dL, glucose: 90 mg/dL, and ADA: 10.7 IU/L). Bacterial and tuberculosis cultures of the fluid showed no evidence of proliferation, and the pathology report ruled out malignancy. Echocardiography did not reveal any pericardial effusions. After 3 months, a quotidian fever (with a daily recurrence) ranging from 39°C to 40°C, which did not respond to antibiotic treatment, commenced. In the examinations; Brucella, EBV, COVID‐19, hepatitis, and HIV tests were negative. Tumor marker tests, including CEA, CA 19‐9, CA 123, and CA 153, as well as serum rheumatologic tests, yielded negative results. Furthermore, the patient underwent colonoscopy and endoscopy, and no malignant findings were discovered. A PET/CT scan showed severe hypermetabolic mediastinal and cervical lymphadenopathies, intense FDG uptake in the spleen and bone marrow medullary areas, and pleural effusions on both sides, with the left side being more prominent.

**FIGURE 2:**
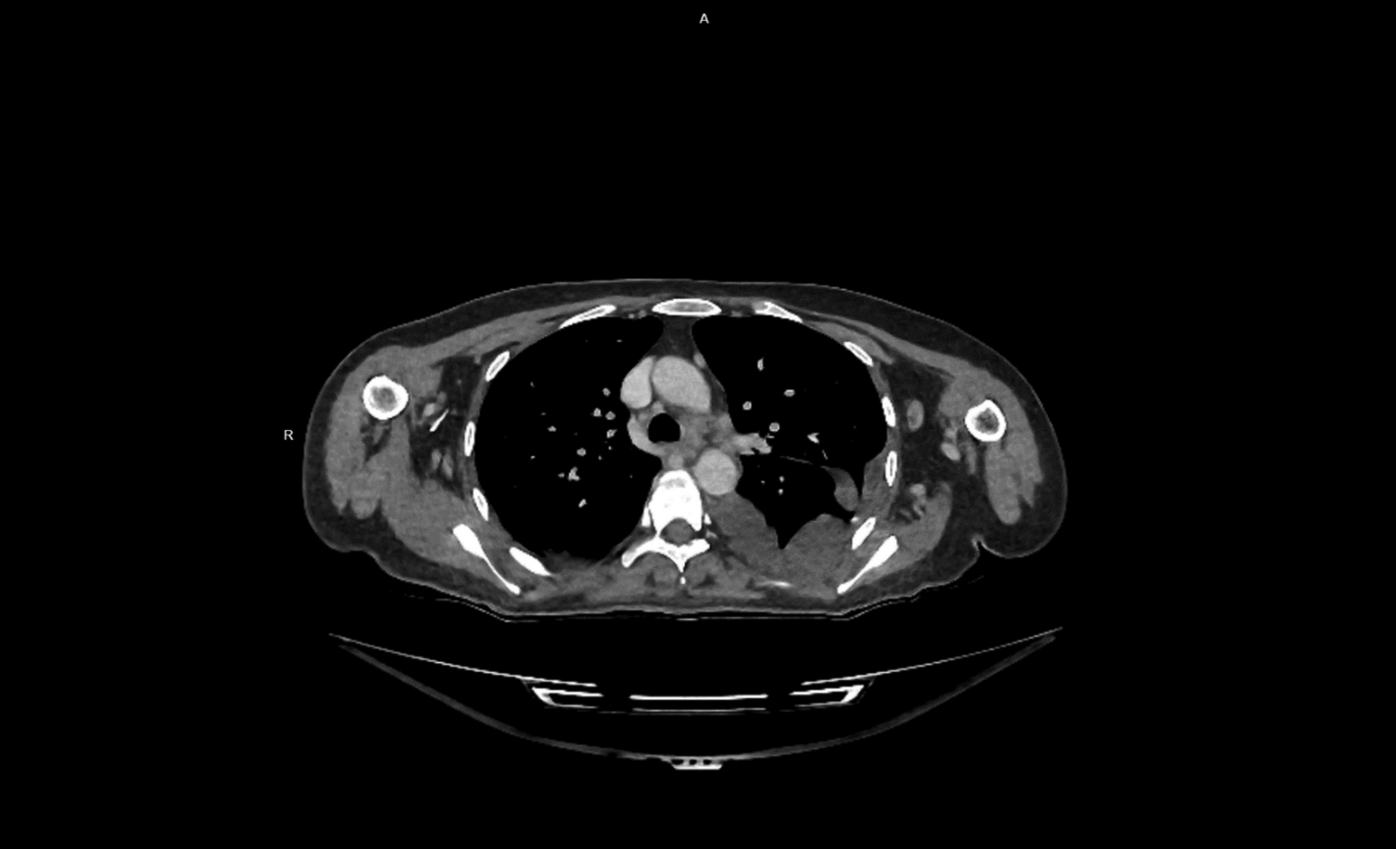
Thorax CT showing mediastinal lymphadenopathies.

The patient, who continued to experience fever, general malaise, and cough, was admitted to our hospital in May 2022. During the physical examination, the patient's overall condition was assessed as moderate, with an alert and cooperative demeanor. Vital signs were found to be within normal ranges. In the examination of the respiratory system, decreased breath sounds were observed in the left lung. The examination of other systems did not reveal any abnormalities and appeared normal. Thoracic CT scan revealed bilateral pleural effusion with more fluid on the left side, and mediastinal and hilar lymphadenopathies (Figure 2). The abdominal CT scan revealed hepatomegaly and splenomegaly. Based on the patient's recent prolonged hospitalization Meropenem, IV fluids, and supportive therapy were started. After hospitalization; the fever was 39°C, and blood cultures and urine cultures were negative. The laboratory test results were as follows: WBC levels of 16.28 K/μL, HB 6.4 g/dL, PLT 533 K/μL, neutrophil 15.12 K/μL (92%), AST 128 U/L, ALT 50 U/L, CRP 260.28 mg/L, procalcitonin 1.55 ng/mL, LDH 727 U/L, ferritin 3254,10 ng/mL, sedimentation 98 mm, COVID‐19 PCR (−), D‐dimer 4.85 μg/mL. Thoracentesis was performed, and fluid analyses revealed: Total protein of 3.9 g/dL, LDH 305 U/L, albumin 1.6 g/dL, and glucose 90 mg/dL. The cytological examination of the pleural fluid unveiled a low levels of lymphocytes and a presence of reactive mesothelial cells, while the subsequent fluid cultures yielded negative results.

Furthermore, a bone marrow biopsy was performed due to spleen and bone marrow involvement in Pet‐CT, and no pathological finding was observed. Rheumatological tests, vasculitis tests, and QuantiFERON tests were all negative.

**FIGURE 3:**
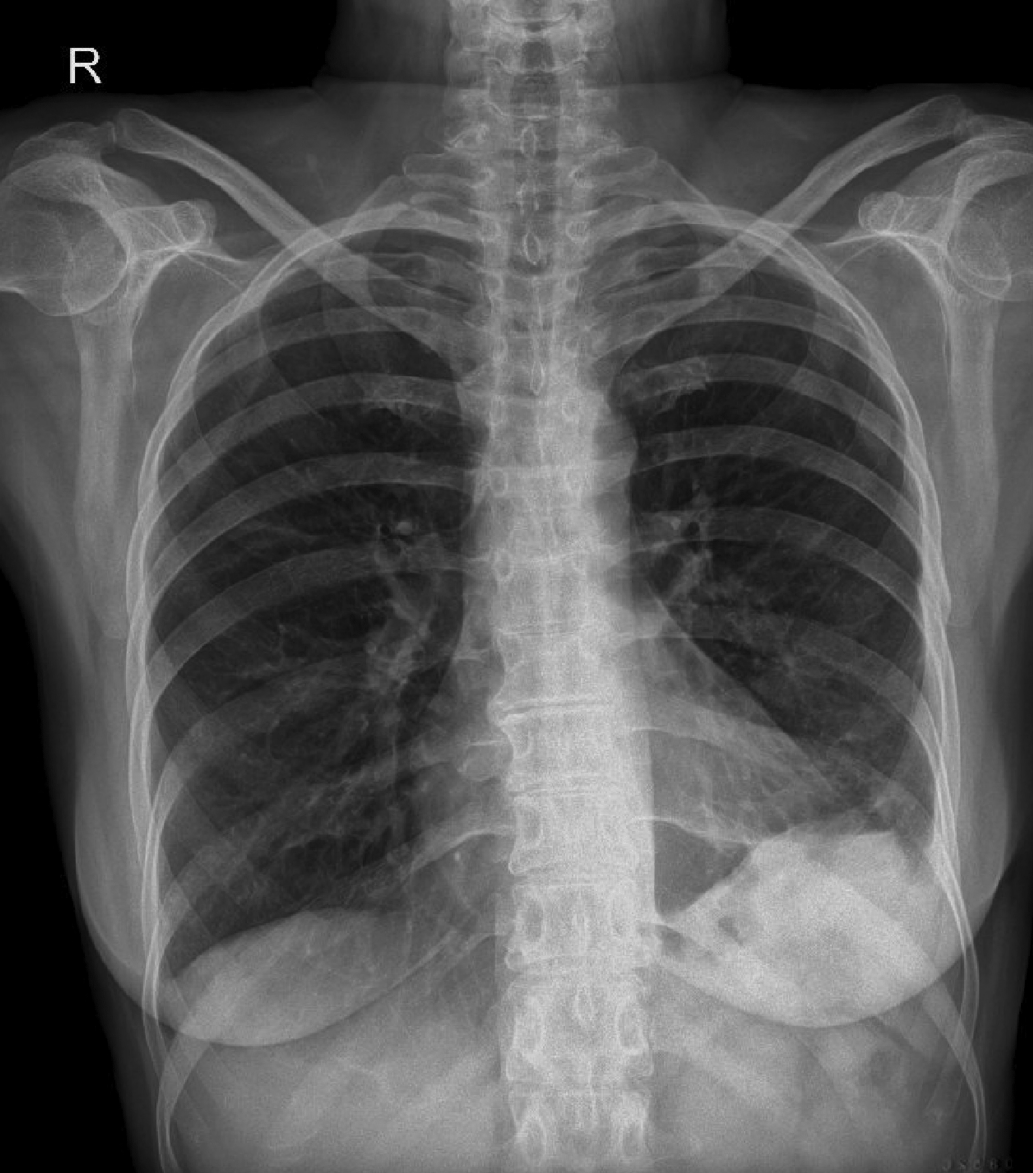
Regression of pleural effusion after starting steroid treatment.

To obtain further diagnostic information, a pleural biopsy using video‐assisted thoracoscopic surgery (VATS) was performed by a thoracic surgeon, and the pathology report indicated chronic nonspecific pleuritis. As a result of our findings, the patient was started on methylprednisolone 1.5 mg/kg/day with the diagnosis of AOSD. After starting steroid treatment, her fever started to decrease, and pleural effusion regressed (Figure 3). After 1 month of methylprednisolone treatment, the patient's therapy was augmented by the addition of methotrexate (MTX) at a weekly dosage of 15 mg. However, after 2 months of treatment, as there was no significant decrease in inflammation markers (CRP 110.9 mg/L, sedimentation rate 98 mm), the patient's treatment was further adjusted by introducing etanercept at a weekly dosage of 50 mg. The patient's current treatment regimen consists of etanercept 50 mg/week, MTX 22.5 mg/week, and methylprednisolone 8 mg/day. Following this treatment protocol, a remarkable improvement has been observed in clinical, and laboratory parameters (CRP 2.5 mg/L, sedimentation rate 4 mm, ferritin 270 ng/mL), indicating near‐complete regression.

## 3 DISCUSSION

AOSD is a systemic inflammatory disorder with no recognized etiology. This disease usually affects young people, with a bimodal peak between the ages of 15–25 and 36–46.^1^ However, after the age of 60, an old age onset has also been reported.^7^, ^8^, ^9^ Our patient falls into the less common late‐onset group.

Many conditions can present with a combination of AOSD‐like symptoms, such as fever, rash, arthritis, lymphadenopathy, elevated acute phase reactants, leukocytosis, and abnormalities in liver enzymes.^10^ A fever of unknown origin (FUO) also has a wide differential diagnosis and should be considered in the differential diagnosis of AOSD.^10^

We ruled out infection etiologies with many tests and cultures. All connective tissue diseases and vasculitis tests were negative. We eliminated malignancy etiologies with negative tumor markers and other pathological and histological examinations. Finally, according to the Yamaguchi criteria (major criteria: fever for 2 months and granulocytic leukocytosis; minor criteria: sore throat, lymphadenopathy, hepatomegaly, splenomegaly, abnormal LFTs, and RF and ANA negative tests), the patient was diagnosed with AOSD.

An elevated ferritin level is a serologic measure of disease activity and a sign of how well a treatment is working.^11^ Ferritin levels can be elevated in infections, malignancies, and liver diseases However, under these circumstances, serum ferritin concentrations hardly ever go above >3000 μg/L values.^12^ Elevated ferritin levels play a major diagnostic tool in the still's disease as in our case with ferritin levels of 3254, 10 ng/mL.

Our case first presented with fever, cough, and pleural effusion. The most typical clinical sign of pulmonary involvement in AOSD is pleurisy, which can be present at the time of initial presentations or more frequently with an acute exacerbation of the condition.^13^

Pleural effusion is usually in a small amount and parenchymal lung involvement (PLI) only occurs in 5% of cases.^14^ These conditions can mimic an infectious disease and this should be kept in mind for differential diagnosis.

As the first‐line therapy, NSAIDs and corticosteroids are recommended and our patient responded well to steroid therapy.

Literature suggests that a pro‐inflammatory cascade may be involved, although our understanding of the processes underlying the pathophysiology of AOSD is largely speculative.^15^ Prominent danger signals like pathogen‐associated molecular patterns (PAMPs) or damage‐associated molecular patterns (DAMPs) are likely where the pro‐inflammatory cascade begins^16^ activation of the innate immune system and overproduction of mature IL‐1β leads to overproduction of several pro‐inflammatory cytokines including IL‐6, IL‐8, IL‐17, IL‐18, and TNF.^15^, ^17^, ^18^ In addition to amplification pathways, regulatory or anti‐inflammatory mechanisms may be lacking or ineffective in the pathophysiology of autoinflammatory illnesses like AOSD.^16^

Our case had a car accident 1 month before pleural effusions and 3 months before the beginning of fever and cough. Following stress and traumatic injury, individuals show altered immunological balance, according to biomarker studies.^19^ Particularly, blood levels of immune‐stimulating Th1 and inflammatory Th17 cells as well as plasma levels of pro‐inflammatory cytokines like IFN‐γ, IL‐6, TNF‐α, and IL‐17 surged, suggesting a pro‐inflammatory condition.^20^ However, further reductions in anti‐inflammatory cytokines and cells have also been seen. Therefore, it can be thought that these inflammatory cytokines were induced by stress and trauma and contributed to the development of Still's disease in our patient. There are no previous reports in the literature for patients with post‐traumatic pleurisy and diagnosed with AOSD.

## 4 CONCLUSION

In conclusion, AOSD is a rare and challenging systemic inflammatory condition with diverse clinical features. Diagnosis can be delayed, and the Yamaguchi criteria are the most sensitive for identifying AOSD. Pleural effusions may serve as a defining characteristic in individuals who meet the diagnostic criteria, and elevated ferritin levels can be a valuable diagnostic marker. Early initiation of steroid therapy is recommended as the first‐line treatment. However, further research is necessary to enhance our understanding of AOSD and advance its management strategies.

## AUTHOR CONTRIBUTIONS

**Feride Yaman:** Conceptualization; data curation; project administration; supervision; validation; writing – review and editing. **Ali Kimiaei:** Conceptualization; formal analysis; investigation; methodology; resources; software; validation; visualization; writing – original draft; writing – review and editing.

## FUNDING INFORMATION

The authors have not received any funding for this paper.

## CONFLICT OF INTEREST STATEMENT

The authors and their close relatives, as well as the research foundations to which they are affiliated, have received no financial payments or other benefits from any commercial entities related to the subject of this article.

## ETHICS STATEMENT

Not applicable.

## CONSENT

Written informed consent to publish this case report was obtained from the patient.

## Data Availability

Not applicable.
